# Artificial liver support system therapy in acute-on-chronic hepatitis B liver failure: Classification and regression tree analysis

**DOI:** 10.1038/s41598-019-53029-0

**Published:** 2019-11-11

**Authors:** Kaizhou Huang, Feiyang Ji, Zhongyang Xie, Daxian Wu, Xiaowei Xu, Hainv Gao, Xiaoxi Ouyang, Lanlan Xiao, Menghao Zhou, Danhua Zhu, Lanjuan Li

**Affiliations:** 1 State Key Laboratory for Diagnosis and Treatment of Infectious Diseases, National Clinical Research Center for Infectious Diseases, Collaborative Innovation Center for Diagnosis and Treatment of Infectious Diseases, The First Affiliated Hospital of Zhejiang University, College of Medicine, Zhejiang University, Hangzhou, Zhejiang Province China; 2 Shulan Hangzhou Hospital, Shulan Health, Hangzhou, Zhejiang Province China

**Keywords:** Hepatitis B, Hepatitis B, Experimental models of disease

## Abstract

Artificial liver support systems (ALSS) are widely used to treat patients with hepatitis B virus-related acute-on-chronic liver failure (HBV-ACLF). The aims of the present study were to investigate the subgroups of patients with HBV-ACLF who may benefit from ALSS therapy, and the relevant patient-specific factors. 489 ALSS-treated HBV-ACLF patients were enrolled, and served as derivation and validation cohorts for classification and regression tree (CART) analysis. CART analysis identified three factors prognostic of survival: hepatic encephalopathy (HE), prothrombin time (PT), and total bilirubin (TBil) level; and two distinct risk groups: low (28-day mortality 10.2–39.5%) and high risk (63.8–91.1%). The CART model showed that patients lacking HE and with a PT ≤ 27.8 s and a TBil level ≤455 μmol/L experienced less 28-day mortality after ALSS therapy. For HBV-ACLF patients with HE and a PT > 27.8 s, mortality remained high after such therapy. Patients lacking HE with a PT ≤ 27.8 s and TBil level ≤ 455 μmol/L may benefit markedly from ALSS therapy. For HBV-ACLF patients at high risk, unnecessary ALSS therapy should be avoided. The CART model is a novel user-friendly tool for screening HBV-ACLF patient eligibility for ALSS therapy, and will aid clinicians via ACLF risk stratification and therapeutic guidance.

## Introduction

Acute-on-chronic liver failure (ACLF) is a complicated syndrome that can cause rapid deterioration in patients with chronic liver disease, associated with high-level mortality^1^. Several large, prospective multicentre studies have shown that patients with ACLF have extremely bad prognoses; the 28-day mortality rate ranges from 30% to 90%^2–4^. In Asian, Hepatitis B virus (HBV) infection accounts for the majority of ACLF^3^. Recently, new diagnostic criteria for HBV-ACLF and a prognostic scoring system were developed in a prospective work conducted by the Chinese Group on the Study of Severe Hepatitis B-ACLF (COSSH-ACLF) (1322 patients in 13 liver centres were studied)^4^. Liver transplantation (LT) effectively treats HBV-ACLF patients who respond poorly to standard treatment, but is limited by organ scarcity. Over the past three decades, artificial liver support systems (ALSS) have been employed to treat liver failure. Previous studies found that ALSS improved short-term survival in those with acute-on-chronic liver failure^5,6^. Some studies, including a prospective controlled study, found that ALSS was safe, well tolerated, and a useful bridge to LT in patients with ACLF^7–9^. However, another study found that HBV-ACLF patients with lower Model for End-stage Liver Disease scores (MELDs) enjoyed significantly better outcomes than did those with higher MELDs^10^. Other studies suggested that ALSS afforded survival benefits in specific groups^11,12^. Thus, subgroups of patients who can benefit from HBV-ACLF, and factors affecting survival, must be identified. To guide and optimise targeted therapy for HBV-ACLF patients, a practical, accurate decision-making tool is urgently needed to help physicians evaluate risks and decide whether to initiate ALSS therapy or to prefer conservative treatment.

Unlike multivariable logistic regression, classification and regression tree (CART) analysis is a non-parametric algorithm based on recursive partitioning. CART analysis separates all values using a decision tree featuring progressive binary splits. Several splits serve as predictors identifying patients at different degrees of risk. Given its convenience and clinical utility, CART analysis has been used to develop predictive models aiding clinical decision-making in various medical fields^13–15^. To help clinicians identify and screen patients eligible for ALSS therapy, we developed an accurate, user-friendly, bedside prognostic model employing CART analysis. We compared the accuracy of our model in term of predicting 28-day mortality to that of a new Z logistic regression model (LRM-Z) and certain older prognostic models, including the model for end-stage liver disease (MELD), integrated model for end-stage liver disease (iMELD), Chronic Liver Failure Consortium acute-on-chronic liver failure (CLIF-C ACLF) score and Chinese group on the Study of Severe Hepatitis B-acute-on-chronic liver failure (COSSH-ACLF) score.

## Results

### Baseline characteristics of the derivation and validation cohorts

**Figure 1:**
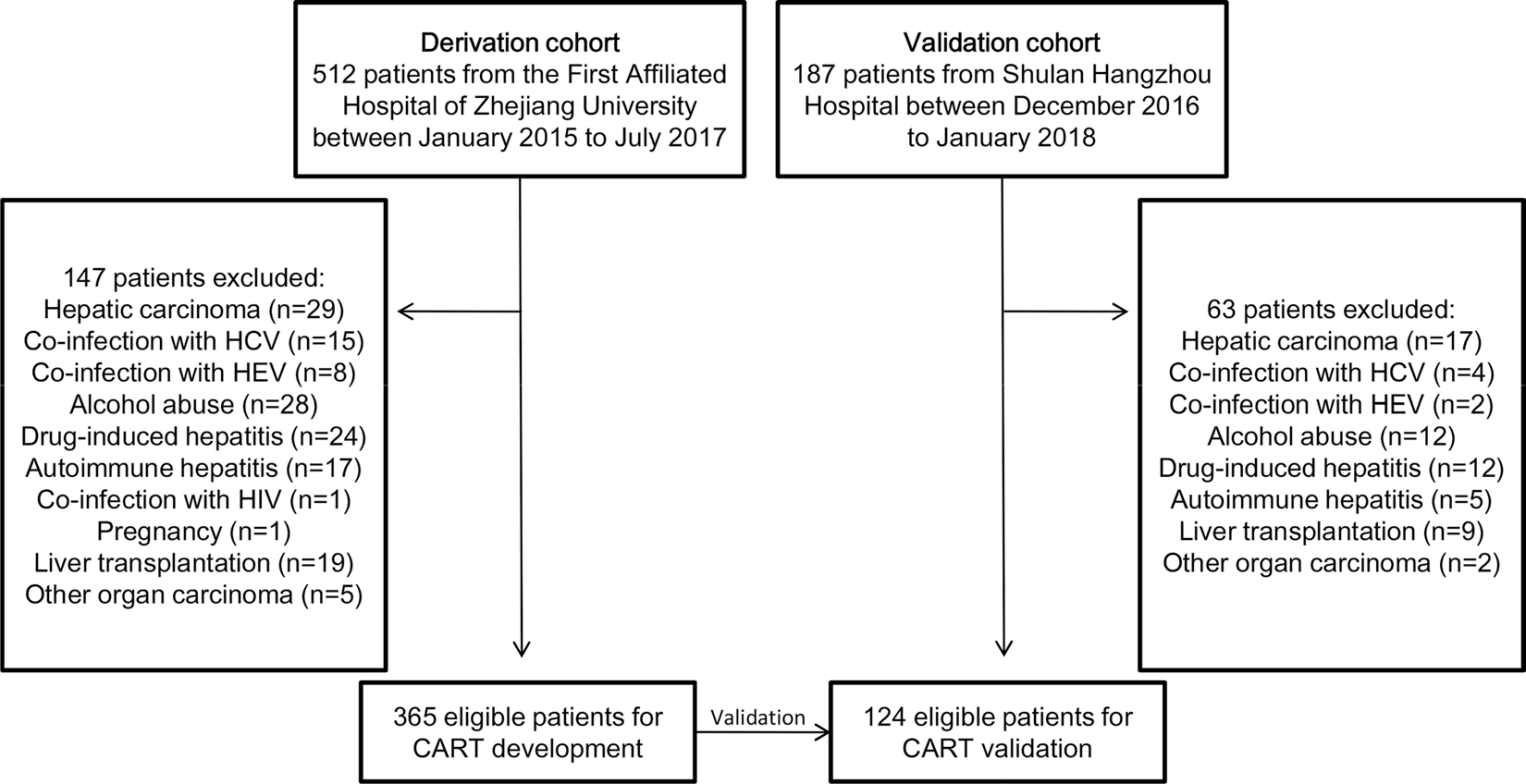
A flow diagram of study participants included in the study.

A total of 699 hospitalised HBV-ACLF patients were initially screened and enrolled; 365 patients in the derivation cohort and 124 in the validation cohort were ultimately included (Fig. 1). The baseline characteristics of both cohorts of patients are listed in Table 1 and Supplementary Table 1. Most patients in the derivation cohort were male and had lower rates of liver cirrhosis (*P* < 0.05). The derivation cohort had lower ALB, glucose, BUN and NH3 levels, and a lower WBC count, than the validation cohort, which in turn had lower D-dimer, ALP, Hb and Plt levels than the deviation cohort (all *P* < 0.05). No significant differences in terms of model scores or mortality were evident between the two cohorts. The characteristics of the derivation and validation cohorts stratified by 28-day mortality are shown in Table 2. The cohorts were similar in terms of the variables significantly influencing survival, i.e. the HE proportion; WBC count; the levels of ALT, TBil, INR, PT, fibrinogen, D-dimer, Hb and NH3; and the scores on all systems tested (all *P* < 0.05). No significant differences were found between ALSS sessions and mortality in both two cohorts (Supplementary Table 2).

**Table 1:** Baseline characteristics of derivation and validation cohorts.

Variable	Derivation Cohort (n = 365)	Validation Cohort (n = 124)	*P*-value
***Clinical parameters***			
Age (years)	46.52 ± 11.38	45.31 ± 11.97	0.314
Male Gender (%)	325 (89.04%)	99 (79.84%)	0.009
HTN (%)	43 (11.78%)	17 (13.71%)	0.572
DM (%)	28 (7.67%)	16 (12.90%)	0.101
Liver Cirrhosis (%)	157 (43.01%)	77 (62.10%)	<0.001
Ascites (%)	166 (45.48%)	68 (54.84%)	0.071
Hepatic Encephalopathy (%)	47 (12.88%)	16 (12.90%)	0.996
Hepatic Encephalopathy I-II (%)	27 (7.40%)	9 (7.26%)	
Hepatic Encephalopathy III-IV (%)	20 (5.48%)	7 (5.65%)	
Infection (%)	59 (16.16%)	29 (23.39%)	0.070
Gastrointestinal hemorrhage (%)	10 (2.74%)	8 (6.45%)	0.580
28 days-Mortality	140 (38.36%)	51 (41.13%)	0.585
***Laboratory parameters***			
ALT (U/L)	182.00 (91.50, 371.50)	155.5 (59.25, 445.75)	0.376
AST (U/L)	138.00 (91.00, 252.00)	143.50 (65.25, 404.50)	0.782
TBil (μmol/L)	424.87 ± 123.14	402.11 ± 132.01	0.082
ALB (g/L)	32.19 ± 6.40	33.75 ± 4.48	0.012
GGT (U/L)	70.00 (50.50, 92.50)	70.00 (43.50, 119.00)	0.706
Glucose (mmol/L)	3.96 (3.27, 4.72)	6.05 (4.27, 8.70)	<0.001
INR	2.11 (1.76, 2.78)	2.28 (1.80, 3.25)	0.054
PT (s)	23.4 (19.8, 30.7)	23.35 (20.03, 35.25)	0.209
Fibrinogen (g/L)	1.21 (0.96, 1.55)	1.29 (0.96, 1.71)	0.294
D-dimer (μg/L)	2279.00 (1120.00, 3690.00)	1318.00 (562.00, 3004.50)	<0.001
ALP (U/L)	130.00 (108.50, 161.00)	118.50 (95.00, 153.75)	0.006
WBC (10^9^/L)	8.04 ± 4.13	9.10 ± 5.40	0.023
Hb (g/L)	126.04 ± 19.73	115.24 ± 28.43	<0.001
Plt (10^9/L)	108.18 ± 48.80	95.73 ± 53.82	0.017
Serum Sodium (mmol/L)	136.79 ± 4.53	137.29 ± 9.09	0.428
Creatinine (μmol/L)	67.00 (58.50, 80.00)	63.50 (49.00, 95.00)	0.071
GFR (ml/min)	106.83 (92.79, 116.36)	111.58 (100.92, 116.09)	0.090
BUN (mmol/L)	4.30 (3.30, 5.80)	5.00 (3.40, 8.48)	0.003
NH3 (μmol/L)	67.00 (50.00, 91.50)	80.70 (51.00, 118.00)	0.002
HBV-DNA (log10, IU/ml)	5.04 ± 2.23	4.61 ± 1.81	0.055
***Scoring system***			
MELD score	25.40 ± 5.55	26.17 ± 7.54	0.297
iMELD score	43.60 ± 7.59	43.66 ± 10.69	0.953
CLIF-C ACLF score	41.17 ± 7.00	41.89 ± 7.39	0.327
COSSH-ACLF score	6.34 (5.83, 6.96)	6.59 (5.91, 7.52)	0.056

Abbreviations: HTN, hypertension; DM, diabetes mellitus; ALT, alanine aminotransferase; AST, aspartate aminotransferase; TBil, total bilirubin; ALB, albumin; GGT, gamma-glutamyl transpeptidase; INR, internationalized normal ration; PT, prothrombin time; ALP, alkaline phosphatase; WBC, white blood cell; Hb, hemoglobin; Plt, platelet; GFR, glomerular filtration rate; BUN, urea nitrogen; HBV-DNA, hepatitis B virus- deoxyribonucleic acid; MELD, Model for End-stage Liver Disease; iMELD, integrated model for end-stage liver disease; CLIF-C ACLF, Chronic Liver Failure Consortium acute-on-chronic liver failure; COSSH-ACLF, Chinese group on the Study of Severe Hepatitis B-acute-on-chronic liver failure.

**Table 2:** Baseline characteristics of derivation and validation cohorts, stratified by 28 day-mortality.

Variable	Derivation Cohort (n = 365)			Validation Cohort (n = 124)		
	Survival (n = 225)	Death (n = 140)	P-value	Survival (n = 73)	Death (n = 51)	*P*-value
***Clinical parameters***						
Age (years)	46.48 ± 11.48	46.57 ± 11.27	0.940	44.04 ± 12.57	47.12 ± 10.91	0.160
Male Gender (%)	199 (88.44%)	126 (88.57%)	0.644	56 (76.71%)	43 (84.31%)	0.299
HTN (%)	27 (12.00%)	16 (11.43%)	0.869	8 (10.96%)	9 (17.65%)	0.287
DM (%)	17 (7.56%)	11 (7.86%)	0.916	10 (13.70%)	6 (11.76%)	0.752
Liver Cirrhosis (%)	94 (41.78%)	63 (45.00%)	0.546	48 (65.75%)	29 (56.86%)	0.315
Ascites (%)	105 (46.67%)	61 (43.57%)	0.564	40 (54.79%)	28 (54.90%)	0.991
Hepatic Encephalopathy (%)	6 (2.67%)	41 (29.29%)	<0.001	2 (2.74%)	14 (27.45%)	<0.001
Hepatic Encephalopathy I-II (%)	2 (0.89%)	25 (17.86%)		2 (2.74%)	7 (13.73%)	
Hepatic Encephalopathy III-IV (%)	4 (1.78%)	16 (11.43%)		0 (0.00%)	7 (13.73%)	
Infection (%)	43 (19.11%)	16 (11.43%)	0.055	14 (19.18%)	15 (20.55%)	0.185
Gastrointestinal hemorrhage (%)	7 (3.11%)	3 (2.14%)	0.584	4 (5.48%)	4 (7.84%)	0.557
sessions	2.14 ± 0.99	1.94 ± 1.04	0.064	2.16 ± 1.00	2.04 ± 1.11	0.514
***Laboratory parameters***						
ALT (U/L)	145.00 (81.50, 284.50)	268.50 (126.25, 542.50)	<0.001	109.00 (38.50, 394.50)	295.00 (103.00, 769.00)	0.002
AST (U/L)	120.00 (84.00, 208.00)	176.50 (104.25, 336.00)	<0.001	113.00 (57.00, 335.50)	240.00 (95.00, 491.00)	0.069
TBil (μmol/L)	404.12 ± 112.48	458.23 ± 132.29	<0.001	376.25±118.29	439.14 ± 142.64	0.009
ALB (g/L)	32.12 ± 7.64	32.30 ± 3.62	0.792	33.58±4.96	34.00 ± 3.72	0.595
GGT (U/L)	66.00 (47.00, 86.50)	75.50 (56.00, 101.00)	0.011	78.00 (46.50, 128.50)	68.00 (41.00, 108.00)	0.439
Glucose (mmol/L)	3.92 (3.28, 4.59)	4.06 (3.12, 5.46)	0.430	6.14 (4.45, 8.72)	5.96 (4.19, 8.41)	0.713
INR	1.95 (1.69, 2.31)	2.67 (2.08, 3.46)	<0.001	1.91 (1.72, 2.57)	3.01 (2.24, 4.30)	<0.001
PT (s)	21.70 (18.85, 25.40)	30.55 (23.05, 37.88)	<0.001	21.60 (19.10, 27.20)	32.50 (22.70, 45.70)	<0.001
Fibrinogen (g/L)	1.31 (1.04, 1.61)	1.08 (0.87, 1.40)	<0.001	1.42 (1.07, 1.83)	1.20 (0.74, 1.46)	0.002
D-dimer (μg/L)	2042.00 (1012.50, 3245.00)	2586.50 (1690.50, 4359.50)	0.001	972.00 (434.00, 2724.50)	1998.00 (1056.00, 3132.00)	0.013
ALP (U/L)	127.00 (106.00, 155.50)	133.50 (114.25, 167.75)	0.030	116.00 (93.50, 155.00)	120.00 (95.00, 149.00)	0.855
WBC (10^9^/L)	7.12 ± 4.05	9.52 ± 3.84	<0.001	8.28 ± 5.07	10.26 ± 5.71	0.050
Hb (g/L)	122.53 ± 18.91	131.69 ± 19.78	<0.001	109.96 ± 26.49	122.80 ± 29.64	0.015
Plt (10^9^/L)	108.23 ± 47.98	108.09 ± 50.27	0.979	105.52 ± 59.52	81.71 ± 41.04	0.015
Serum Sodium (mmol/L)	137.00 ± 4.17	136.46 ± 5.07	0.284	137.96 ± 5.08	136.33 ± 12.83	0.394
Creatinine (μmol/L)	67.00 (59.00, 80.00)	66.00 (57.25, 83.50)	0.732	60.00 (49.50, 92.50)	69.00 (41.00, 100.00)	0.739
GFR (ml/min)	107.58 (94.45, 116.36)	106.38 (90.09, 116.21)	0.632	111.58 (102.72, 118.30)	111.58 (88.45, 113.01)	0.129
BUN (mmol/L)	4.00 (3.25, 5.40)	5.00 (3.30, 6.73)	<0.001	4.70 (3.35, 9.24)	5.61 (3.41, 8.30)	0.631
NH3 (μmol/L)	65.00 (48.00, 84.50)	73.00 (53.75, 110.25)	0.002	69.00 (46.00, 90.00)	103.00 (71.10, 170.00)	<0.001
HBV-DNA (log10, IU/ml)	4.86 ± 2.23	5.32 ± 2.20	0.051	4.62 ± 1.96	4.60 ± 1.60	0.942
***Scoring system***						
MELD score	23.72 ± 4.08	28.09 ± 6.49	<0.001	24.21 ± 6.67	28.98 ± 7.87	<0.001
iMELD score	41.76 ± 6.42	46.54 ± 8.38	<0.001	40.85 ± 9.53	47.69 ± 11.07	<0.001
CLIF-C ACLF score	38.89 ± 6.26	44.83 ± 6.58	<0.001	39.36 ± 7.10	45.51 ± 6.27	<0.001
COSSH-ACLF score	6.03 (5.67, 6.51)	6.90 (6.39, 7.62)	<0.001	6.11 (5.49, 6.88)	7.50 (6.65, 8.09)	<0.001

Abbreviations: HTN, hypertension; DM, diabetes mellitus; ALT, alanine aminotransferase; AST, aspartate aminotransferase; TBil, total bilirubin; ALB, albumin; GGT, gamma-glutamyl transpeptidase; INR, internationalized normal ration; PT, prothrombin time; ALP, alkaline phosphatase; WBC, white blood cell; Hb, hemoglobin; Plt, platelet; GFR, glomerular filtration rate; BUN, urea nitrogen; HBV-DNA, hepatitis B virus- deoxyribonucleic acid; MELD, Model for End-stage Liver Disease; iMELD, integrated model for end-stage liver disease; CLIF-C ACLF, Chronic Liver Failure Consortium acute-on-chronic liver failure; COSSH-ACLF, Chinese group on the Study of Severe Hepatitis B-acute-on-chronic liver failure.

### CART and LRM-Z analysis in the derivation cohort

**Figure 2:**
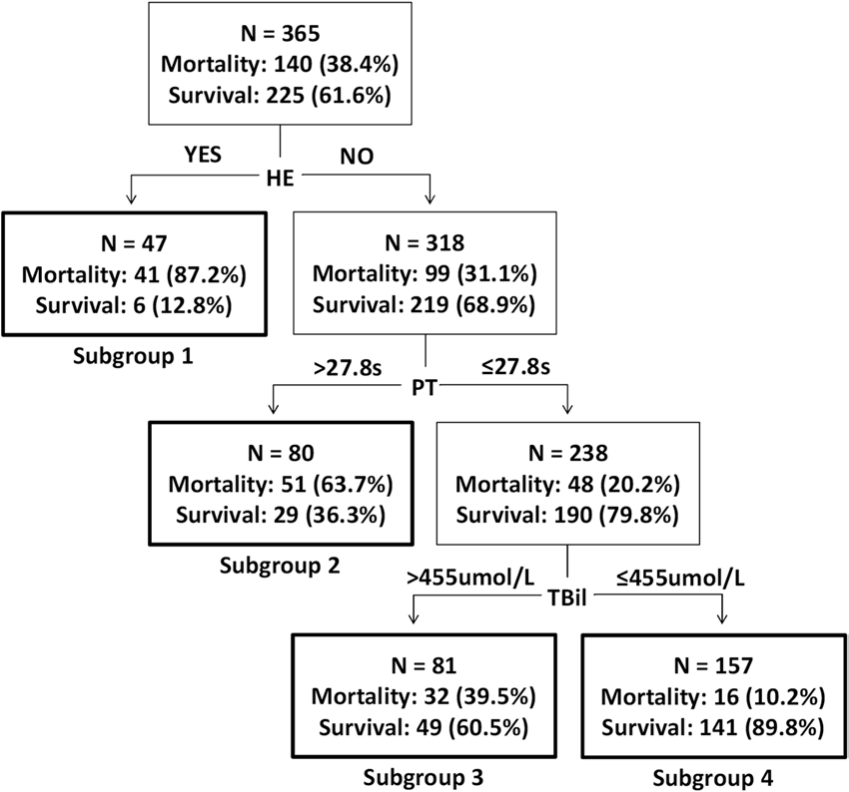
Predictors of ALSS therapy on HBV-ACLF patients and risk stratification for the derivation cohort.

In CART analysis, HE status served as the initial variable. After HE separation, a PT of 27.8 s was chosen as the second split variable in patients lacking HE. When the PT ≤ 27.8 s, the next best predictor was the TBil level, at an optimal cut-off of 455 μmol/L. No additional node afforded any increment in risk discrimination. Therefore, four subgroups of patients, differing significantly in terms of 28-day mortality, were generated by three predictive variables identified via CART analysis: subgroup 1 (patients with HE); subgroup 2 (patients lacking HE but with a PT > 27.8 s); subgroup 3 (patients lacking HE and with a PT ≤ 27.8 s, but a TBil level > 455 μmol/L); and subgroup 4 (patients lacking HE with a PT ≤ 27.8 s, and a TBil level ≤ 455 μmol/L) (Fig. 2). Subgroups 1 and 2 were combined to form a high-risk group, with mortality rates ranging from 63.7 to 87.2%, whereas subgroups 3 and 4 were combined to form a low-risk group, with mortality rates ranging from 10.2 to 39.5%. HBV-ACLF patients in the high-risk group exhibited a 9.883-fold (95% CI: 6.002–16.275-fold, *P* < 0.001) increased in 28-day mortality (compared to those in the low-risk group) after ALSS-treatment (Table 3). Detailed descriptions of LRM-Z analysis are given in the Supplemental Results and Supplementary Table 3.

**Table 3:** 28-day mortality between risk groups.

Group	Derivation cohort			P-value	Validation cohort			*P*-value
	No. of subjects (%)	Mortality (%)	OR (95% CI)		No. of subjects (%)	Mortality (%)	OR (95% CI)	
Low Risk	238 (65.21)	50 (21.00)	—	—	74 (59.68)	16 (21.62)	—	—
High Risk	127 (34.79)	92 (72.44)	9.883 (6.002–16.275)	<0.001	50 (40.32)	35 (70.00)	8.485 (3.726–19.202)	<0.001
Total	365 (100.00)	142 (38.90)	2.394 (1.644–3.488)	<0.001	124 (100.00)	51 (41.13)	2.533 (1.310–4.895)	<0.001

### Validation and comparison

CART analysis was validated for its efficiency of risk stratification efficiency in an independent validation cohort containing 124 subjects. Using the flow chart of the classification tree, each patient was allocated to a subgroup (Supplementary Fig. 1). All patients were also stratified into low- and high-risk groups. Compared to those in the low-risk group, patients in the high-risk group exhibited an 8.485-fold (95% CI: 3.726–19.202-fold, *P* < 0.001) increase in 28-day mortality, similar to what was found in the derivation cohort (Table 3).

**Figure 3:**
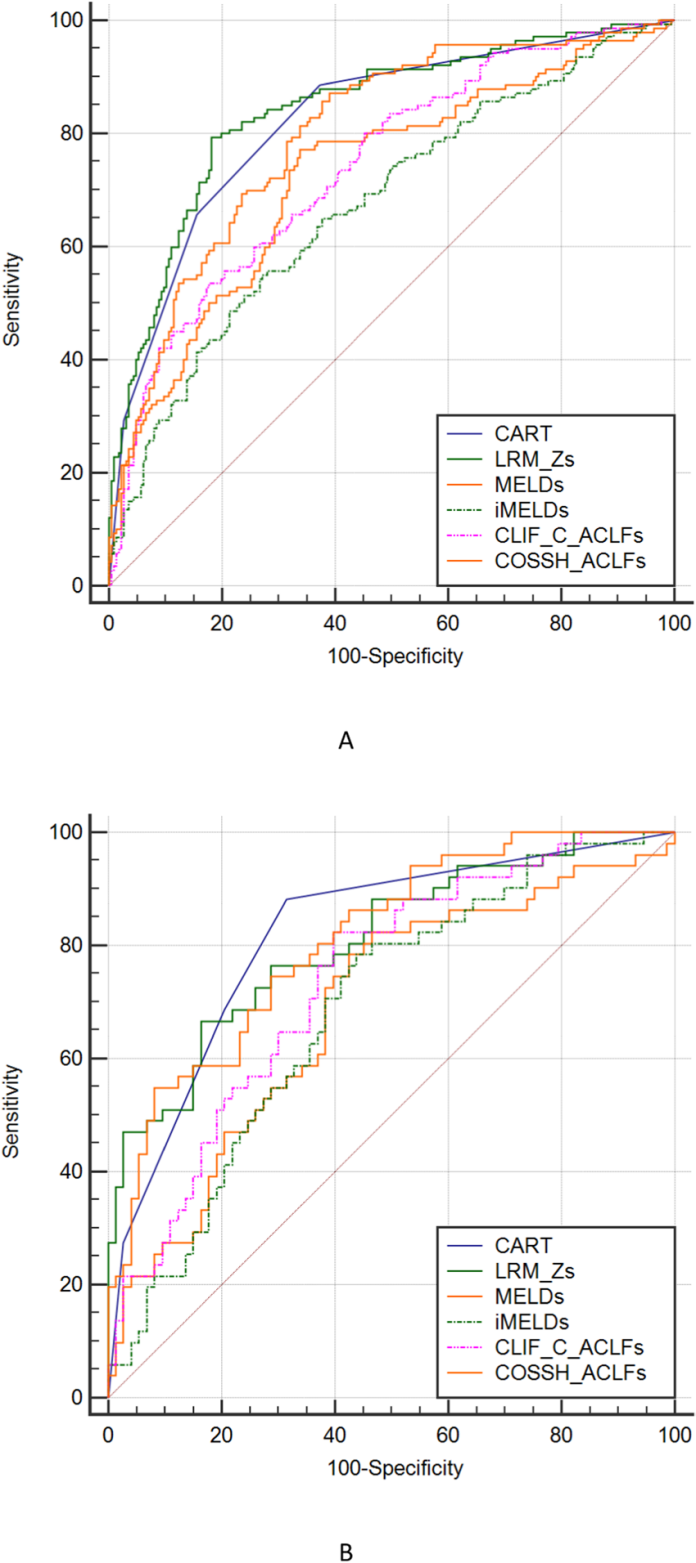
ROC analysis of the predictive accuracy of CART model, LRM-Z, MELD, iMELD, CLIF-C ACLF and COSSH-ACLF score to predict 28-day mortality of acute-on-chronic hepatitis B liver failure in derivation cohort (**A**) and validation cohort (**B**).

The predictive power in terms of 28-day mortality of ALSS-treated HBV-ACLF patients was compared among the CART, LRM-Z and some earlier prognostic models (Fig. 3). In the derivation cohort (Fig. 3A), CART analysis afforded high performance, with an auROC of 0.824 (95% CI: 0.781–0.862). LRM-Z and COSSH-ACLF afforded similar accuracies, with auROCs of 0.842 (95% CI: 0.800–0.878, *P* = 0.1919) and 0.800 (95% CI: 0.755–0.840, *P* = 0.1746), respectively. The MELD, iMELD and CLIF-C ACLF had lower auROCs than the CART, 0.727 (95% CI: 0.678–0.772, *P* = 0.0001), 0.675 (95% CI: 0.625–0.723, *P* < 0.0001) and 0.742 (95% CI: 0.694–0.786, *P* = 0.0011) respectively (Table 4, Supplementary Table 4).

**Table 4:** The predictive value of mortality of the CART score and other models in the derivation and validation cohorts.

Models	auROC	Derivation cohort	*P*-value	auROC	Validation cohort	P-value
		95% CI			95% CI	
CART	0.824	0.781–0.862	—	0.820	0.741–0.883	—
LRM-Z	0.842	0.800–0.878	0.1919	0.807	0.727–0.873	0.6621
MELD	0.727	0.678–0.772	0.0001	0.686	0.597–0.766	0.0070
iMELD	0.675	0.625–0.723	<0.0001	0.685	0.596–0.766	0.0152
CLIF-C ACLF	0.742	0.694–0.786	0.0011	0.738	0.651–0.812	0.0985
COSSH-ACLF	0.800	0.755–0.840	0.1746	0.810	0.730–0.875	0.7523

Abbreviations: CART, classification and regression tree; LRM-Z, logistic regression model Z; MELD, Model for End-stage Liver Disease; iMELD, integrated model for end-stage liver disease; CLIF-C ACLF, Chronic Liver Failure Consortium acute-on-chronic liver failure; COSSH-ACLF, Chinese group on the Study of Severe Hepatitis B-acute-on-chronic liver failure.

In validation (Fig. 3B), CART analysis featured the highest auROC, 0.820 (95% CI: 0.741–0.883), and afforded better performance with higher statistical significance than MELD (0.686, 95% CI: 0.597–0.766, *P* = 0.0070) and iMELD (0.685, 95% CI: 0.596–0.766, *P* = 0.0152). Although statistical significance was not attained, LRM-Z, CLIF-C ACLF and COSSH-ACLF all had lower auROCs [0.807 (95% CI: 0.727–0.873, *P* = 0.6621), 0.738 (95% CI: 0.651–0.812, *P* = 0.0985), and 0.810 (95% CI: 0.730–0.875, *P* = 0.7523), respectively] than CART analysis.

## Discussion

ACLF is one of the most intractable clinical problems worldwide, characterised by severe hepatic abnormalities and rapid disease progression^16^. The short-term mortality rate of HBV-ACLF is extremely high; it is essential to stratify patients by their current condition and possible prognosis to select appropriate treatment strategies^17^. Liver transplantation is optimal, but is compromised by donor organ scarcity and the need to select patients carefully^18,19^. ALSS therapies have been considered useful to replace liver function, affording an opportunity for hepatic recovery or stabilising the clinical status prior to transplantation^20^. However, the optimal timing of ALSS treatment and the target population remain remains unclear. Clinicians are in urgent need of a better method to correctly identify patients that would benefit from such treatment, to avoid unnecessary clinical burdens^21^. It is essential to screen patients with ACLF and in terms of factors that would allow them to benefit from ALSS therapy. Here, we established and validated a CART approach toward analysis and identification of predictive factors in subgroups of patients with HBV-ACLF who would benefit from ALSS therapy.

HE is the most common complication of HBV-ACLF. Previous studies reported that HE in hospitalised ACLF patients was associated with a high mortality rate^22^. Another study indicated that HE obviously affected the clinical prognosis of such patients^23^. Here, LRM-Z confirmed that HE was independently prognostic of 28-day mortality. In CART analysis, HE was the first variable split. HBV-ACLF patients with HE were allocated to subgroup 1; the 28-day mortalities were 87.2 and 87.5% in the derivation and validation cohorts, respectively. The high 28-day mortality rate of subgroup 1 suggests that HBV-ACLF patients with HE might have difficulty in earning benefits from ALSS therapy. It is generally accepted that hyperbilirubinaemia and coagulopathy are the two most prominent features of liver failure, as indicated by both official criteria and studies on ACLF diagnosis and prognosis worldwide^2,3,24,25^. PT and the TBil level were positively associated with mortality risk in both CART and LRM-Z analyses. PT was the second variable split in CART analysis and was also an independent prognostic factor in the LRM-Z model, suggesting a positive correlation between PT and ACLF mortality, consistent with data from previous studies^26,27^. It is also well received for TBil level to be the third split variable. Our results are in line with previous studies reporting that the TBil level was independently prognostic of ACLF^28,29^. CART analysis also stratified subjects into low- and high-risk groups that exhibited significant differences. Patients in the low-risk group exhibited lower than usual 28-day mortality compared to most patients with HBV-ACLF^4^, strongly indicating that ALSS therapy may improve overall survival in such patients. However, patients in the high-risk group may be difficult to earn benefits from ALSS therapy; LT is required as soon as possible.

Compared to traditional multivariate models, CART analysis has unique advantages. First, CART analysis can process high-dimensional data (even highly skewed data) when the sample size is low^30^. CART analysis can calculate probabilities and impurities using the non-missing values; missed values are ignored^31^. From our results, CART is very comparable to models using logistic regression in terms of predictive value of mortality. However, models constructed with the aid of various logistic regression coefficients are very complex, and clinical utility is compromised. Given its simple classification parameters and cut-off values, the CART model is simple and user-friendly in the hands of clinicians. Third, the CART analysis stratified patients into low- and high-risk groups exhibiting significant differences. Thus, the model facilitates subgroup/risk stratification in a manner similar to how clinicians make decisions, which may improve the management of hospitalised patients with HBV-ACLF. Patients at lower risk can be reassured that ALSS therapy will play a positive and active role in their treatment programs, but unnecessary ALSS therapy should be avoided, and LT prioritised in patients at higher risk.

Our study had certain limitations. First, potential confounders in small data samples may cause the significance of included risk factors to be overestimated, thus influencing the actual risk, which may explain why the prognostic factors identified by the CART model and LRM-Z differed^32^. Further work with a larger population is needed. Second, the CART was built in a single centre and validated in another centre, using homogeneous data. The multicentre adaptability and feasibility of CART requires further verification. Third, only HBV-infected patients were included. To generalise its use, the CART model requires further validation in patients with ACLF of diverse aetiologies. Thus, multicentre, prospective studies with larger patient populations are needed to further verify the applicability of our model.

However, the CART model is a novel, validated, user-friendly bedside tool that can screen HBV-ACLF patients in terms of eligibility for ALSS therapy. HBV-ACLF patients lacking HE and with a PT ≤ 27.8 s may benefit from such therapy. In this group, the benefit may be more pronounced when the TBil level ≤ 455 µmol/L. The CART model helps physicians correctly identify patients at lower risk (facilitating appropriate ALSS use as part of a treatment program), and to prioritise liver transplantation for patients at higher risk.

## Methods

### Patients

Patients were screened at two different medical centres operating identical medical record systems. Medical data prior to ALSS therapy were collected from patient records, and derivation and validation cohorts were defined (derivation cohort: the First Affiliated Hospital of Zhejiang University [patients treated between January 2015 and July 2017]; validation cohort: Shulan Hangzhou Hospital [patients treated between December 2016 and January 2018]). The study protocol conformed to the ethical guidelines of the 1975 Declaration of Helsinki and was approved by the Clinical Research Ethics Committees of the First Affiliated Hospital, Zhejiang University School of Medicine, and Shulan Hangzhou Hospital. Written informed consent was obtained from all patients or their legal surrogates prior to enrolment.

### Inclusion and exclusion criteria

The enrolment criteria for the patients with HBV-ACLF corresponded to the COSSH-ACLF^4^, which was developed to diagnose HBV-ACLF specifically. Briefly, HBV-ACLF was defined as acute deterioration of liver function and/or extrahepatic organ failure in patients underlying HBV-related chronic liver disease regardless of cirrhosis status. Detailed descriptions of the inclusion and exclusion criteria are given in the Supplemental Methods.

### Diagnostic criteria of complications

Liver cirrhosis was diagnosed based on symptoms and signs of portal hypertension and findings on ultrasonography, computed tomography or magnetic resonance imaging. Ascites was confirmed via paracentesis, abdominal imaging and other clinical evidence. HE assessment and grading employed the West Haven criteria^33^. Gastrointestinal haemorrhage was diagnosed by a positive faecal occult blood test or the presence of blood in vomit. Infections included spontaneous bacterial peritonitis, pulmonary infections and urinary tract infections, and were explored via imaging and laboratory culture^34^. Organ failure was diagnosed using the chronic liver failure-sequential organ failure assessment (CLIF-SOFA) score^2^.

### Treatments

#### Standard medical therapy

All patients received standard medical therapy including bed rest, adequate nutritional support and single or combination of antiviral drugs. Sodium restriction, diuretics and paracentesis combined with albumin administration was used for ascites; Patients with hepatic encephalopathy received lactulose and L-ornithine aspartate; Appropriate antibiotics were applied for infections and adjusted based on the laboratory culture; Gastrointestinal haemorrhage were treated with somatostatin, pituitrin, proton pump inhibitors and necessary endoscopic therapy.

#### ALSS therapy

All patients received uniformed plasma exchange (PE) plus plasma bilirubin adsorption (PBA) ALSS therapy. For patients with HE, plasma perfusion (PP) was used as part of the ALSS therapy regimen^10^. The total exchanged plasma volume was 2500–3500 mL, with the exchange rate was 20–25 mL/min. The flow rate of blood was 100–130 ml/min. Dexamethasone (5 mg) and heparin (2500 U) were injected routinely before ALSS therapy. Protamine sulphate (20–50 mg) was used for neutralization in every session. Each session of ALSS therapy lasted for 4–6 hours and was repeated every 2–4 days. ALSS therapy was discontinued when the overall improvement in the patient’s status and TB < 200 µmol/L or conditions such as bleeding and circulatory abnormalities that did not allow further ALSS therapy^35^. A total of 752 sessions (average 2 sessions/patient, ranging from 1 to 5 sessions) of ALSS therapy in derivation cohort, while a total of 262 sessions (average 2 sessions/patient, ranging from 1 to 4 sessions) of ALSS therapy were performed in validation cohort (Supplementary Table 5).

ALSS therapy was initiated within 2 days of admission. The date of diagnosis of HBV-ACLF was the follow-up commencement date.

### Data collection

We collected data on patient demographics and complications, laboratory measurements of alanine aminotransferase (ALT), aspartate aminotransferase (AST), TBil, albumin, gamma-glutamyl transpeptidase(GGT), sodium, and glucose; the INR; prothrombin time (PT); the levels of fibrinogen, D-dimer, alkaline phosphatase (ALP), and creatinine; the glomerular filtration rate (GFR); the levels of urea nitrogen (BUN), NH3, hepatitis B virus- deoxyribonucleic acid (HBV-DNA), white blood cell (WBC), hemoglobin (Hb) and platelet count (Plt). All laboratory data were collected at the time of hospital admission or prior to ALSS therapy.

The scores of published prognostic models (MELD, iMELD, CLIF-C ACLF and COSSH-ACLF) were calculated using the following formulas:

$$\begin{array}{c}{\rm{MELD}}\,{\rm{score}}=9.6\times \,\mathrm{ln}({\rm{Creatinine}}[{\rm{mg}}/{\rm{dL}}]/88)+3.8\times \,\mathrm{ln}({\rm{TBil}}[{\rm{umol}}/{\rm{L}}]/18)\\ \,\,\,\,\,\,\,+\,11.2\times \,\mathrm{ln}({\rm{INR}})+6.4;\end{array}$$

$${\rm{iMELD}}\,{\rm{score}}={\rm{MELD}}+0.3\times {\rm{Age}}[{\rm{years}}]-0.7\times {\rm{Na}}+[{\rm{mmol}}/{\rm{L}}]+100$$

$$\begin{array}{c}{\rm{CLIF}}-C\,\mathrm{ACLF}\,{\rm{score}}=10\times (0.33\times {\rm{CLIF}}-{\rm{OFs}}+0.04\times {\rm{Age}}[{\rm{years}}]\\ \,\,\,\,\,\,\,\,\,\,\,\,+\,0.63\times \,\mathrm{ln}({\rm{WBC}}\,{\rm{counts}}[{10}^{9}])-2)\end{array}$$

$$\begin{array}{c}{\rm{COSSH}}-\mathrm{ACLF}\,\mathrm{score}=0.741\times {\rm{INR}}+0.523\times {\rm{HBV}}-{\rm{SOFA}}\\ \,\,\,\,\,\,\,\,\,\,\,+\,0.026\times {\rm{Age}}[{\rm{years}}]+0.003\times {\rm{TBil}}[{\rm{umol}}/{\rm{L}}].\end{array}$$

### Construction of the CART and LRM-Z

Using selected variables, CART analysis divided all data into two homologous groups exhibiting different survival outcomes; the best splits and cut-off values were derived for each variable^4,36–38^. Then, the algorithm allocated data by reference to the best overall split of all best splits to a parent node, which then produce two child nodes exhibiting higher homogeneities. This process was repeated using both tree-building and -pruning until statistical analysis indicated that no further reduction in node impurity was possible or that pre-specified stop criteria had been met^39^. This generated several subgroups with predicted mortality rates. We used CART analysis to identify and screen HBV-ACLF patients eligible for ALSS therapy. Two risk groups were identified base on the mortality rates of subgroups. The ability of CART to stratify ALSS-treated HBV-ACLF patients into subgroups by mortality risks was tested using the independent validation cohort. Patients from this cohort were allocated to subgroups using the flow chart of the CART tree. Mortality was calculated for each subgroup, and odds ratios (ORs) and 95% confidence interval (CI) were calculated when risk groups were compared in both cohorts. Detailed descriptions of the LRM-Z construction are given in the Supplemental Methods.

### Statistical analysis

Normality of distribution was explored for all variables. Continuous variables that were normally distributed were expressed as mean ± standard deviations and other variables as median with interquartile ranges. Categorical variables are expressed as percentages and counts. Student’s *t*-test or the Mann–Whitney U-test was used to compare continuous variables. The Pearson chi-squared test or Fisher’s exact test was employed to compare categorical variables and proportions between groups, as appropriate. A *P*-value < 0.05 was considered statistically significant. The capacities of various scoring systems to differentiate survivors from non-survivors were assessed by evaluating areas under receiver operating characteristic curves (auROCs). CART analysis was performed with the C50 package in R version 3.4.3 (http://www.r-project.org/). Other statistical analyses was performed using SPSS ver. 18.0 software (SPSS Inc., Chicago, IL, USA). ROC curves were drawn with the aid of MedCalc ver. 18.2 software (Mariakerke, Belgium).

## Supplementary information

Supplementary Info
