# Supplementary material for: Artificial liver support system therapy in acute-on-chronic hepatitis B liver failure: Classification and regression tree analysis
Source: Sci Rep. 2019 Nov 11;9:16462. doi: 10.1038/s41598-019-53029-0 (PMC6848208; doi:10.1038/s41598-019-53029-0)
Supplement: Supplementary file 1 — Supplementary Info [file 41598_2019_53029_MOESM1_ESM.docx]

**Supplementary Material**

**Artificial liver support system therapy in acute-on-chronic hepatitis B liver failure: Classification and regression tree analysis**

Kaizhou Huang^1#^, Feiyang Ji^1#^, Zhongyang Xie^1#^, Daxian Wu^1^, Xiaowei Xu^1^, Hainv Gao^2^, Xiaoxi Ouyang^1^, Lanlan Xiao^1^, Menghao Zhou^1^, Danhua Zhu^1^, Lanjuan Li^1*^

*** Corresponding Author:** Lanjuan Li, The First Affiliated Hospital of Zhejiang University, College of Medicine, Zhejiang University, No.79 Qingchun Road, Shangcheng District, 310003, Hangzhou, Zhejiang Province, China

E-mail: ljli@zju.edu.cn

Telephone: +86-571-87236458

Fax: +86-571-87236459

**Contents of Supplementary data**

| **Contents** | **Number** |
| --- | --- |
| Supplementary Methods | 2 |
| Supplementary Results | 1 |
| Supplementary Figures | 1 |
| Supplementary Tables | 5 |

**Supplementary Methods**

1. **Inclusion and exclusion criteria**

Inclusion criteria were:

1. patients aged between 16 and 80 years;
2. patients with HBV-related chronic liver disease, according to the 2009 AASLD guidelines:

positive for the HBV surface antigen ≥6 months; serum HBV-DNA ≥20000 IU/mL (10^5^ copies/mL); persistently or intermittently elevated alanine aminotransferase/aspartate aminotransferase levels and a liver biopsy showing chronic hepatitis.

1. patients were diagnosed as ACLF, according to the COSSH-ACLF criteria:
2. patients with kidney failure alone;
3. patients with single liver failure (TB≥205 μmol/L or 12 mg/dL) with an INR ≥1.5;
4. patients with single liver failure and kidney dysfunction and/or hepatic encephalopathy;
5. patients with single type of organ failure of the coagulation, circulatory or respiratory systems and kidney dysfunction and/or hepatic encephalopathy;
6. patients with cerebral failure alone plus kidney dysfunction;
7. patients with failures of more than two organ systems.
8. patients who received ALSS therapy for at least one time.

Exclusion criteria included the following:

1. hepatic carcinoma;
2. co-infection with human hepatitis A, C, D or E viruses;
3. co-infection with immunodeficiency virus;
4. alcoholic abuse;
5. drug-induced hepatitis;
6. autoimmune hepatitis;
7. liver transplantation;
8. patients with other organ carcinoma.
9. **Construction of the LRM-Z**

We used the LRM-Z validate the effectiveness of CART analysis in terms of the prognoses of the derivation cohort. Then, outcome variables that differed significantly (*P* < 0.05) between patients according to univariate logistic regression were subjected to multivariate logistic regression to identify independent prognostic predictors for the ALSS-treated HBV-ACLF patients. In this analysis, the conditional probabilities for stepwise factor entry and removal were 0.05 and 0.10, respectively. ORs and 95% CIs were calculated as well.

***Abbreviations:*** *HBV: hepatitis B virus; AASLD: American Association for the Study of Liver Diseases;* *HBV-DNA, hepatitis B virus- deoxyribonucleic acid; COSSH-ACLF, Chinese group on the Study of Severe Hepatitis B-acute-on-chronic liver failure; TBil: total bilirubin; INR: internationalised normal ratio; ALSS: artificial liver support system; CART, classification and regression tree; LRM-Z, logistic regression model Z.*

**Supplementary Results**

1. **Construction of the LRM-Z**

On univariate logistic regression analysis in the derivation cohort (Supplementary Table 3), we found that HE; the levels of ALT, AST, TBil, glucose, INR, fibrinogen, D-dimer, ALP, Hb, BUN and NH3; the PT; and the WBC count were all significantly associated with 28-day mortality (all *P* < 0.05). These variables were then entered to multivariate logistic regression analysis. In multivariate logistic regression analysis, HE (OR = 2.595, 95% CI: 1.310-5.137, *P* = 0.006); WBC count (OR = 1.104, 95% CI: 1.029-1.186, *P* = 0.006); PT (OR = 1.136, 95% CI: 1.093-1.180, *P* < 0.001); and the levels of TBil (OR = 1.003, 95% CI: 1.001-1.006, *P* = 0.004) and Hb (OR = 1.015, 95% CI: 1.001-1.029, *P*=0.035) were all independent predictors of mortality. Finally, using the regression coefficients of the five independent variables associated with 28-day mortality as revealed by multivariate logistic regression, a new prognostic model (LRM-Z) for ALSS-treated HBV-ACLF patients was derived using the following formula:

$$LRM-Z=0.953\times HE[no HE, yes=1; HE I-II, yes=2; HE III-IV, yes=3]+0.127\times PT[s]+0.003\times TBil[umol/L]+0.099\times WBC counts[10^9]+0.015\times Hb[g/L]-9.042.$$

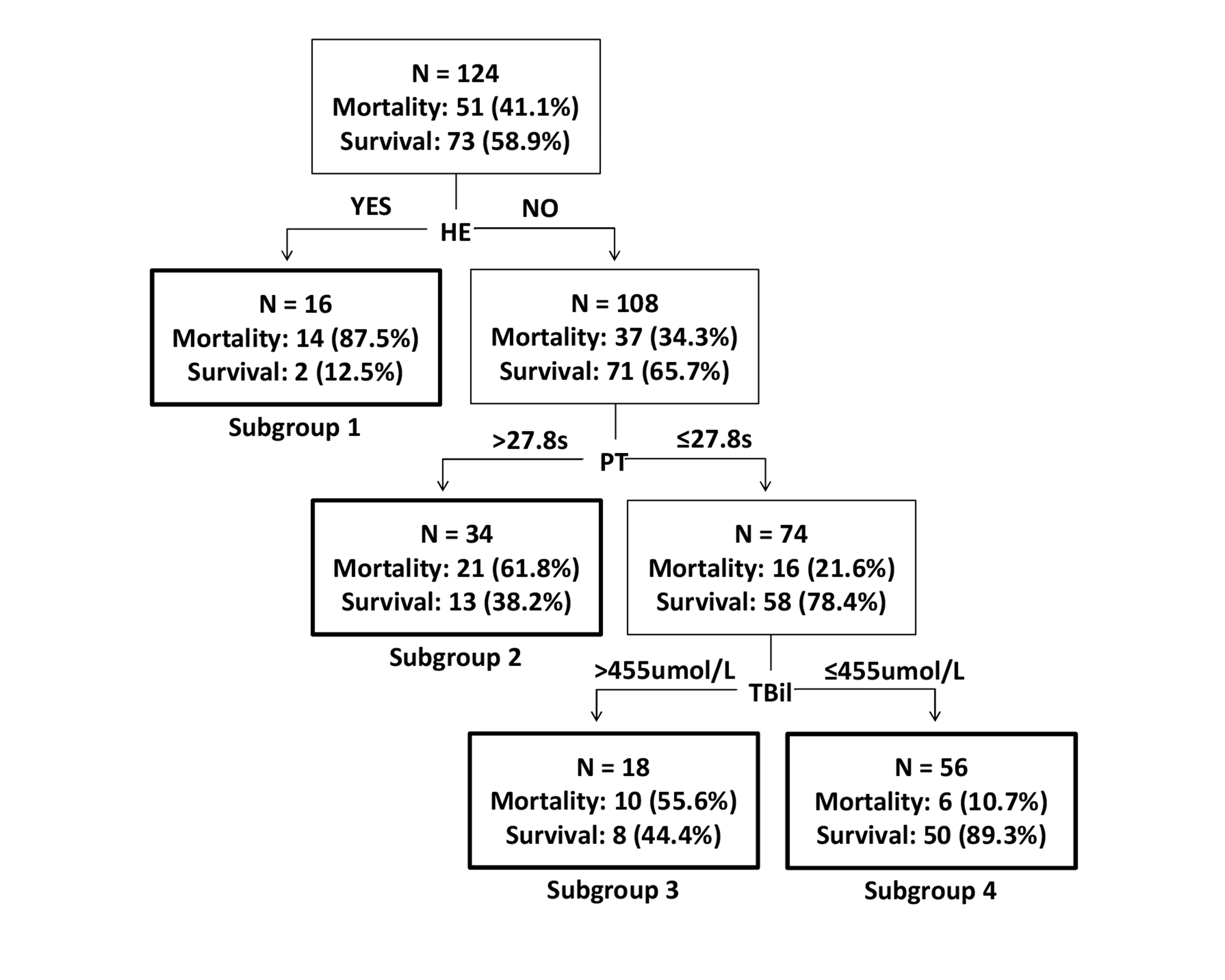


**Supplementary Figure 1.** Predictors of ALSS therapy on HBV-ACLF patients and risk stratification for the validation cohort

***Abbreviations:*** *ALSS: artificial liver support system; HBV-ACLF: hepatitis B virus-related acute-on-chronic liver failure; TBil: total bilirubin; PT: prothrombin time; HE: hepatic encephalopathy.*

**Supplementary Table 1**. The number of organ failure and mortality rate in both derivation and validation cohort

| **Number of organ failure** | **28-day Mortality rate** | | ***P*-value** |
| --- | --- | --- | --- |
|  | **Derivation Cohort(n=365)** | **Validation Cohort(n=124)** |  |
| ***1*** | 22.08% (53/240) | 24.59% (15/61) |  |
| ***2*** | 66.34% (67/101) | 50.00% (27/54) | 0.146 |
| ***3 or more*** | 83.33% (20/24) | 100% (9/9) |  |

Data are expressed as percent mortality (number of deaths/total number of patients)

**Supplementary Table 2.** Numbers of ALSS Sessions and survival rate in derivation and validation cohorts

| **Numbers of Sessions** | **Survival rate** | |
| --- | --- | --- |
|  | **Derivation Cohort(n=365)** | **Validation Cohort(n=124)** |
| ***1*** | 53.33% (64/120) | 48.84% (21/43) |
| ***2*** | 64.90% (98/151) | 69.05% (29/42) |
| ***3 or more*** | 67.02% (63/94) | 58.97% (23/39) |
| ***P*-value** | 0.07 | 0.167 |

**Supplementary Table 3.** Univariate and multivariate logistic regression analysis of the associations between 28 day-mortality

and variables in the derivation cohort

| Variable | Univariate analysis | | | | Multivariate analysis | | | |
| --- | --- | --- | --- | --- | --- | --- | --- | --- |
|  | **B** | **OR** | **95%CI** | ***P*-value** | **B** | **OR** | **95%CI** | ***P*-value** |
| *Clinical parameters* |  | | | |  | | | |
| Age (years) | 0.001 | 1.001 | 0.982-1.019 | 0.940 |  |  |  |  |
| Male Gender (%) | 0.162 | 1.176 | 0.592-2.227 | 0.644 |  |  |  |  |
| HTN (%) | -0.055 | 0.946 | 0.490-1.827 | 0.869 |  |  |  |  |
| DM (%) | 0.042 | 1.043 | 0.474-2.298 | 0.916 |  |  |  |  |
| Liver Cirrhosis (%) | 0.131 | 1.140 | 0.745-1.745 | 0.546 |  |  |  |  |
| Ascites (%) | -0.125 | 0.882 | 0.577-1.349 | 0.564 |  |  |  |  |
| Hepatic Encephalopathy (%) | -1.720 | 5.583 | 2.944-10.590 | <0.001 | 0.953 | 2.595 | 1.310-5.137 | 0.006 |
| Infection (%) | -0.605 | 0.546 | 0.294-1.013 | 0.055 |  |  |  |  |
| Gastrointestinal hemorrhage (%) | -0.383 | 0.682 | 0.173-2.682 | 0.584 |  |  |  |  |
| *Laboratory parameters* |  | | | |  | | | |
| ALT (U/L) | 0.002 | 1.002 | 1.001-1.002 | <0.001 |  |  |  |  |
| AST (U/L) | 0.002 | 1.002 | 1.001-1.004 | <0.001 |  |  |  |  |
| TBil (μmol/L) | 0.004 | 1.004 | 1.002-1.005 | <0.001 | 0.003 | 1.003 | 1.001-1.006 | 0.004 |
| ALB (g/L) | 0.004 | 1.004 | 0.972-1.038 | 0.792 |  |  |  |  |
| GGT (U/L) | 0.004 | 1.004 | 1.000-1.008 | 0.075 |  |  |  |  |
| Glucose (mmol/L) | 0.106 | 1.112 | 1.010-1.225 | 0.031 |  |  |  |  |
| INR | 1.466 | 4.332 | 2.968-6.323 | <0.001 |  |  |  |  |
| PT (s) | 0.141 | 1.151 | 1.111-1.193 | <0.001 | 0.127 | 1.136 | 1.093-1.180 | <0.001 |
| Fibrinogen (g/L) | -0.614 | 0.541 | 0.332-0.882 | 0.014 |  |  |  |  |
| D-dimer (μg/L) | 0.000 | 1.000 | 1.000-1.000 | 0.003 |  |  |  |  |
| ALP (U/L) | 0.006 | 1.006 | 1.001-1.011 | 0.014 |  |  |  |  |
| WBC (10^9/L) | 0.174 | 1.190 | 1.115-1.270 | <0.001 | 0.099 | 1.104 | 1.029-1.186 | 0.006 |
| Hb (g/L) | 0.025 | 1.025 | 1.013-1.037 | <0.001 | 0.015 | 1.015 | 1.001-1.029 | 0.035 |
| Plt (10^9/L) | 0.000 | 1.000 | 0.996-1.004 | 0.978 |  |  |  |  |
| Serum Sodium (mmol/L) | -0.028 | 0.972 | 0.926-1.021 | 0.265 |  |  |  |  |
| Creatinine (μmol/L) | 0.005 | 1.005 | 1.000-1.011 | 0.730 |  |  |  |  |
| GFR (ml/min) | -0.006 | 0.994 | 0.985-1.003 | 0.163 |  |  |  |  |
| BUN (mmol/L) | 0.151 | 1.163 | 1.069-1.265 | <0.001 |  |  |  |  |
| NH3 (μmol/L) | 0.012 | 1.012 | 1.006-1.019 | <0.001 |  |  |  |  |
| HBV-DNA (log10,IU/ml) | 0.097 | 1.102 | 0.999-1.215 | 0.053 |  |  |  |  |

***Abbreviations:*** *HTN, hypertension; DM, diabetes mellitus; ALT, alanine aminotransferase; AST, aspartate aminotransferase; TBil, total bilirubin; ALB, albumin; GGT, gamma-glutamyl transpeptidase; INR, internationalized normal ration; PT, prothrombin time; ALP, alkaline phosphatase; WBC, white blood cell; Hb, hemoglobin; Plt, platelet; GFR, glomerular filtration rate; BUN, urea nitrogen; HBV-DNA, hepatitis B virus- deoxyribonucleic acid.*

**Supplementary Table 4.** The predictive value of mortality of the CART model and other models in the derivation and validation cohorts

| Models | Youden Index | Sensitvity(%) | Specifcity(%) | +LR | -LR | +PV | -PV |
| --- | --- | --- | --- | --- | --- | --- | --- |
| Derivation cohort |  |  |  |  |  |  |  |
| CART | 0.5124 | 88.57 | 62.67 | 2.37 | 0.18 | 59.60 | 89.80 |
| LRM-Z | 0.6106 | 79.29 | 81.78 | 4.35 | 0.25 | 73.00 | 86.40 |
| MELD | 0.4337 | 77.14 | 66.22 | 2.28 | 0.35 | 58.70 | 82.30 |
| iMELD | 0.2789 | 55.00 | 72.89 | 2.03 | 0.62 | 55.80 | 72.20 |
| CLIF-C ACLF | 0.3579 | 53.57 | 82.22 | 3.01 | 0.56 | 65.20 | 74.00 |
| COSSH-ACLF | 0.4803 | 87.14 | 69.89 | 2.23 | 0.21 | 58.10 | 88.40 |
| Validation cohort |  |  |  |  |  |  |  |
| CART | 0.5673 | 88.24 | 68.49 | 2.80 | 0.17 | 66.20 | 89.30 |
| LRM-Z | 0.5023 | 66.67 | 83.56 | 4.06 | 0.40 | 73.90 | 78.20 |
| MELD | 0.3597 | 78.43 | 57.53 | 1.85 | 0.37 | 56.30 | 79.20 |
| iMELD | 0.3460 | 78.43 | 56.16 | 1.79 | 0.38 | 55.60 | 78.80 |
| CLIF-C ACLF | 0.4263 | 82.35 | 60.27 | 2.07 | 0.29 | 59.20 | 83.00 |
| COSSH-ACLF | 0.4668 | 54.90 | 91.78 | 6.68 | 0.49 | 82.40 | 74.40 |

***Abbreviations:*** *HTN, hypertension; DM, diabetes mellitus; ALT, alanine aminotransferase; AST, aspartate aminotransferase; TBil, total bilirubin; ALB, albumin; GGT, gamma-glutamyl transpeptidase; INR, internationalized normal ration; PT, prothrombin time; ALP, alkaline phosphatase; WBC, white blood cell; Hb, hemoglobin; Plt, platelet; GFR, glomerular filtration rate; BUN, urea nitrogen; HBV-DNA, hepatitis B virus- deoxyribonucleic acid; CART, classification and regression tree; LRM-Z, logistic regression model Z;MELD, Model for End-stage Liver Disease; iMELD, integrated model for end-stage liver disease; CLIF-C ACLF, Chronic Liver Failure Consortium acute-on-chronic liver failure; COSSH-ACLF, Chinese group on the Study of Severe Hepatitis B-acute-on-chronic liver failure.*

**Supplementary Table 5.** Numbers of ALSS Sessions and patients in derivation and validation cohorts

| **Numbers of Sessions** | **Derivation Cohort(n=365)** | **Validation Cohort(n=124)** |
| --- | --- | --- |
| ***1*** | 120 | 43 |
| ***2*** | 151 | 42 |
| ***3*** | 55 | 21 |
| ***4*** | 30 | 18 |
| ***5*** | 9 | - |
| **Total sessions** | 752 | 262 |
